# Combination of Dexmedetomidine and Low-Dose Ketamine in 4 Sugar Gliders (*Petaurus breviceps*) Undergoing Elective Castration

**DOI:** 10.3390/vetsci12080699

**Published:** 2025-07-25

**Authors:** Elisa Silvia D’Urso, Monia Martorelli, Giulia Bersanetti, Paolo Selleri, Chiara De Gennaro

**Affiliations:** 1 Independent Researcher, 00131 Rome, Italy; 2 Centro Veterinario Specialistico (CVS), Via Sandro Giovannini 51-53, 00137 Rome, Italy; 3 Department of Comparative, Diagnostic and Population Medicine, University of Florida College of Veterinary Medicine, Gainesville, FL 32608, USA; cdegennaro@ufl.edu

**Keywords:** dexmedetomidine, exotic anaesthesia, ketamine, orchiectomy, sugar gliders

## Abstract

Four entire male sugar gliders (*Petaurus breviceps*) belonging to the same colony were presented for elective orchiectomy. After clinical examination, dexmedetomidine (120 μg/kg) in combination with ketamine (5 mg/kg) were administered subcutaneously (SC). Once righting and pedal withdrawal reflexes were lost, ringer lactate solution, enrofloxacin and meloxicam were administered SC and a bilateral intratesticular block with lidocaine 0.25% was performed. Heart, respiratory rates and pulse oximetry values were recorded every minute. Onset of sedation, additional use of isoflurane, duration of anaesthesia, duration of surgery, time of recovery after atipamezole administration, quality of recovery and time of food intake were recorded. Postoperative assessment (posture, level of activity, vocalisation, response to manipulation, attention to the surgical wound) was performed hourly until discharge, five hours after surgery. Dexmedetomidine in combination with ketamine provided adequate short-lasting anaesthesia for castration in 3 out of 4 sugar gliders. One sugar glider needed additional isoflurane administration to perform orchiectomy. No perioperative additional analgesia was needed in any sugar glider.

## Simple Summary

Small mammals are becoming increasingly more popular as house pets. Among these, sugar gliders (*Petaurus breviceps*) are small marsupials referred to veterinary facilities both for routine and emergency check-ups, diagnostics and surgeries. Given their delicate nature and specific physiological needs, anaesthetic management requires careful consideration. In addition, data found in the literature regarding anaesthesia management in this species are scarce. This case series presents a multimodal approach to anaesthesia and pain management in sugar gliders undergoing castration, evaluating both physiological parameters and the influence of individual temperament, which can significantly affect induction and recovery from anaesthesia.

## 1. Introduction

Sugar gliders (Petaurus breviceps) are small nocturnal marsupials native to New Guinea and Australia, whose popularity as house pets is increasing. Given their small size and wild nature, chemical restraint is frequently needed to carry out investigations and perform surgeries. In small mammals, general anaesthesia is usually induced and maintained with halogenated inhalant anaesthetics, such as isoflurane and sevoflurane [1,2,3]. This method is routinely used for sugar gliders in the clinical setting, to achieve rapid induction and recovery [1,2]. However, due to its pungent odour, the use of isoflurane has been associated with apnoea and vomiting in this species [1]. Dose-dependent cardiovascular depression, due to both a direct negative inotropic effect and peripheral vasodilation is reported in several species [4,5,6] as well as dose-dependent respiratory depression and reduction in minute volume ventilation [5]. Moreover, in rabbits, sialorrhea and voluntary apnoea are reported [3,7]; the latter may lead to hypercapnia and respiratory acidosis and may promote marked bradycardia and sudden death [7,8]. In addition, because of the stress induced by restraint and catecholamines release [9], in rabbits, induction with inhalants is no longer recommended [3,7,8,9,10]. To reduce the dose-dependent side effects of inhalant anaesthetics and to provide analgesia, it is advisable to pre-administer sedatives and/or analgesics [2].

Information regarding the use of injectable anaesthetics in sugar gliders is sparse and mostly anecdotical. A combination of midazolam (0.35–0.5 mg/kg) and ketamine (10–50 mg/kg) provides heavy sedation in this species, although the onset and duration have not been investigated [2].

The use of dexmedetomidine, a highly selective alpha-2 adrenoreceptor agonist with sedative, analgesic and muscle relaxant properties [11] has not been reported in sugar gliders.

Analgesic drugs routinely used in sugar gliders are non-steroidal anti-inflammatory drugs (NSAIDs), such as meloxicam and opioids, such as buprenorphine. Buprenorphine, a partial μ opioid receptor agonist that provides mild to moderate analgesia, is usually administered before surgical procedures (up to 0.03 mg/kg SC) and in combination with inhalational anaesthesia [2].

Opioid-sparing and opioid-free anaesthesia have been investigated in recent years both in human and veterinary medicine due to the interest in preventing opioid-related side effects (decreased gastrointestinal motility, urinary retention, hyperalgesia and, in humans, addiction and withdrawal symptoms) [12,13,14,15].

The authors would like to report the use of an opioid-free protocol, with dexmedetomidine and low-dose ketamine to induce and maintain general anaesthesia in 4 sugar gliders undergoing elective castration.

## 2. Materials and Methods

Four entire male sugar gliders (SG1, SG2, SG3 and SG4), aged 20.5 ± 2.5 months, weighting 69.7 ± 10.1 g (Table 1), belonging to the same colony, were presented at our facility to undergo elective castration.

The animals were deemed healthy based on physical examination, behaviour, recent history, food intake and littering. Heart rate (HR) was measured by auscultation using a neonatal stethoscope (Littmann Classic II Infant, Oakdale, CA, USA), while respiratory rate was determined by observing chest movements. Three out of four sugar gliders tolerated handling and allowed gentle physical examination, without displaying signs of distress. The youngest (SG4) showed signs of aggression such as hissing, crabbing and assuming a defensive upright posture. Physical examination was performed with the help of a towel, while chest auscultation was not performed. The owner stated that written consent was obtained after clinical examination.

**Figure 1:**
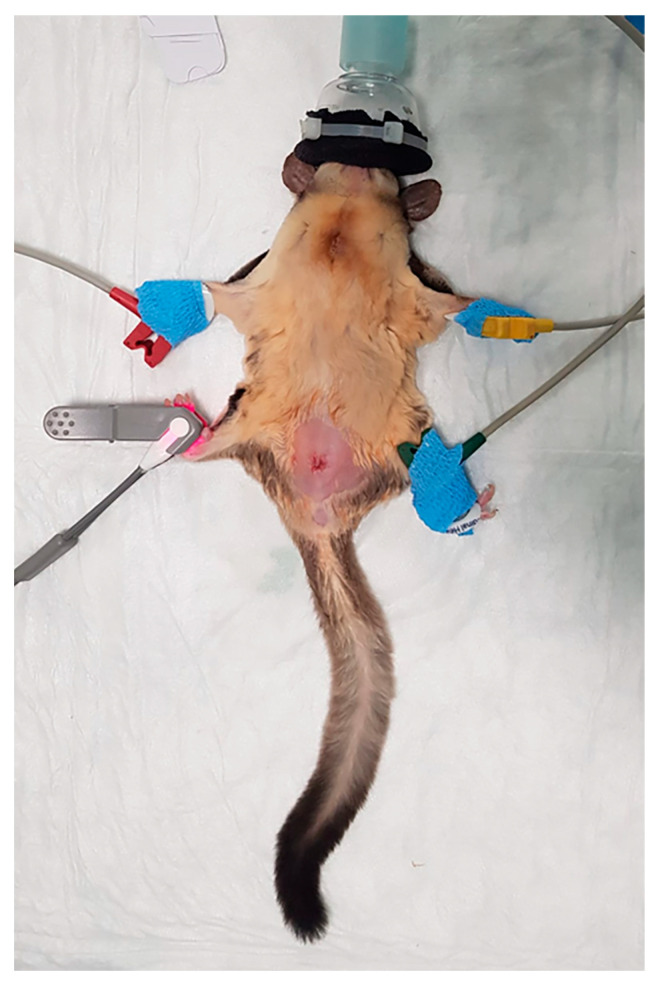
A sugar glider instrumented with SpO_2_ probe and ECG pads (picture taken at the end of the surgery).

All sugar gliders were starved at home for 5 h before surgery [2]. Dexmedetomidine (120 μg/kg) and ketamine (5 mg/kg) were administered SC along the midline of the thorax with a microfine insulin syringe (Insumed 30G, Pic Solution, Como, Italy). The animals were then placed in a small plexiglass chamber saturated with 100% O_2_ at 2 L/min. Once sedated, the animals were removed from the chamber and righting and pedal withdrawal reflexes were assessed. Optimal sedation was defined as a loss of both righting and withdrawal reflexes which were considered our endpoints [16]. Sugar gliders were then positioned in dorsal recumbency on a warm heating pad (Pro heat mat, Swell, UK). Oxygen 100% was delivered via a tightly fitted face mask (Figure 1) with a fresh gas flow of 0.5 mL per minute with a linear, Mapleson D Ayre T-piece throughout the procedure. Balanced electrolytic solution 10 mL/kg (Hartmann’s lactated ringers, B-Braun, Melsugen, Germany), enrofloxacin 5 mg/kg (Baytril, Bayer, Milan, Italy) diluted in saline solution 0.9% and meloxicam 0.2 mg/kg (Metacam, Boehringer Ingelheim, Fornovo San Giovanni, Italy) were administered SC before the procedure [16]. Electrocardiography (ECG) and pulse oximetry (SpO_2_) were monitored via multiparameter monitor (ePM12M Vet, Mindray Animal Medical Technology, Shenzhen, China) and recorded at 1 min intervals (Figure 1). Respiratory rate (RR) was monitored and recorded by observing chest movements at 1 min intervals. Baseline values of HR and RR for each sugar glider were recorded after instrumentation, before clipping the surgical area. A 20% increase in HR or RR compared to baseline was considered a sign of nociception [17], and if present, treated with ketamine 5 mg/kg SC as rescue analgesia. In case of inadequate sedation (persistence of withdrawal or righting reflex), isoflurane was delivered in 100% O_2_ with a fresh gas flow of 0.5 L/min via a tight fitted mask (at a starting concentration of 1.5%) and adjusted throughout the surgery to ensure the absence of purposeful movements. The testicles and surrounding area were clipped and prepped with chlorhexidine 2% solution, the intratesticular block was performed with lidocaine 0.25% (Lidocaina, Ecuphar, Milan, Italy) 0.05 mL per testicle.

Sugar gliders’ castration is described in detail elsewhere [2]. Briefly, orchiectomy and scrotal ablation were performed with a circumferential incision around the pedicle of the scrotum. Each spermatic cord was closed with transfixing ligatures with absorbable 4/0 braided suture material (Vicryl, Ethicon, London, UK) and the testes were removed. The skin incision was closed with a single intradermal monofilament absorbable suture (Monocryl, Ethicon, UK).

At the end of the procedure, atipamezole 0.1 mg/kg (Antisedan, Zoetis, Rome, Italy) was administered subcutaneously (using the same volume as dexmedetomidine). The sugar gliders were allowed to recover in the plexiglass chamber with 100% O_2_ at 2 L/min.

Onset of sedation, additional use of isoflurane, duration of procedure (from SC dexmedetomidine and ketamine injection to atipamezole administration), duration of surgery, time of recovery after atipamezole administration and time of food intake were recorded. Recovery quality was classified as good if the animal returned to cognitive and motor function calmly and uneventfully, or poor if signs of dysphoria were observed. Postoperative evaluations included posture, activity level, vocalisation, response to gentle handling and attention to the surgical site. Rescue analgesia (buprenorphine 0.02 mg/kg SC) was administered if the animal was deemed to be in pain, based on a combination of behavioural and physiological indicators. A postoperative consult was scheduled with the surgeon 7 days after surgery, during which a full physical examination was performed to detect signs of overgrooming or self-injury. Additionally, wound healing was assessed to rule out wound dehiscence or infection.

## 3. Results

Data are reported as mean ± standard deviation. All sugar gliders tolerated SC injections well. The onset of sedation was 7 min ± 3 min and 30 s. One sugar glider (SG4) required isoflurane (1.5%) in 100% oxygen at 0.5 L/min delivered via face mask to reach the loss of righting reflex. Isoflurane (1%) in 100% oxygen at 0.5 L/min was then maintained throughout the procedure. The administration of isoflurane was well tolerated, and no apnoea or vomiting occurred.

The mean HRs recorded after instrumentation (baseline) and surgery were 202 ± 17 and 157 ± 5 beats per minute, respectively. Mean RRs after instrumentation (baseline) and surgery were 24 ± 0.8 and 20 ± 1.6 breaths per minute, respectively. SpO_2_% was equal or above 98% throughout the anaesthesia. In the intraoperative period, no signs of nociception were detected; therefore, rescue analgesia was not administered.

Surgery time was 6 min 15 s ± 54 s and recovery time 4 min ± 2 min 54 s. The overall time of the procedure was 13 min 1 s ± 3 min 1 s. Mean HR, RR, surgery and recovery time for each patient are resumed in Table 1.

All recoveries were calm and uneventful (good quality of recovery), except for SG4 who exhibited signs of dysphoria (compulsive licking of paws, ataxia, lack of coordination, vocalisation) and tried to remove the stitches from the surgical wound (poor quality of recovery). A rigid buster collar was provided to prevent self-traumatism.

All animals started to eat within one hour from the end of the procedure, exhibiting normal gait and posture. SG1, SG2 and SG3 allowed gentle handling without showing signs of discomfort or interest to the surgical site. The rigid buster collar was removed from SG4 one hour after the end of the surgery. Despite still exhibiting fractious behaviour, there was no further attempt to remove the stitches, and handling for hourly assessment was performed using a towel for safe physical restraint. All animals were discharged five hours later with prescribed meloxicam and enrofloxacin PO. Seven days after surgery, body weight and physical examination were unremarkable. The surgical site appeared healed and no signs of general or localised overgrooming or self-injury were present in any patient.

## 4. Discussion

The combination of dexmedetomidine (120 μg/kg) and ketamine (5 mg/kg) administered SC and reversed with atipamezole, provided short-lasting anaesthesia in four sugar gliders undergoing elective orchiectomy. In one of them, anaesthesia provided with this combination was inadequate for surgery, and inhalant anaesthetic administration was required.

To the authors’ knowledge, this is the first reported use of dexmedetomidine for anaesthesia in sugar gliders. In addition to its analgesic, sedative and myorelaxant properties, dexmedetomidine can significantly reduce the minimum alveolar concentration (MAC) of inhalant anaesthetics, dose-dependently, and can be easily reversed administering atipamezole to promote a faster recovery [5]. The reported dose of dexmedetomidine in small mammals ranges from 25 to 500 µg/kg, depending on the species, route of administration and concurrent use of other drugs [2]. The dose selected for this case series was based on three main considerations. First, the high metabolic rate of sugar gliders, which may shorten the duration of sedation. Second, the subcutaneous route of administration, which can delay absorption and peak plasma concentrations compared to intramuscular or intravenous routes. Third, the need to achieve reliable sedation when combined with a low dose of ketamine, in order to minimise the risk of dysphoria following the full reversal of dexmedetomidine.

The combination of ketamine and either medetomidine or dexmedetomidine has been described in cats and rabbits undergoing orchiectomy [15,18]. In cats, the intramuscular administration of ketamine (5 mg/kg) and dexmedetomidine (10 μg/kg) together with an intratesticular lidocaine block provided successful conditions to perform castration without further drugs administration [15]. Furthermore, the reversal with atipamezole significantly reduced recovery times compared to the group of cats receiving ketamine-midazolam-acepromazine and reversed with flumazenil [15]. In rabbits, ketamine (15 mg/kg) and medetomidine (0.25 mg/kg) IM resulted in a faster onset and greater isoflurane-sparing effect compared to ketamine (3 mg/kg) and midazolam [19].

In our case series, 3 out of 4 sugar gliders were successfully anaesthetised with our protocol; however, SG4 required the administration of isoflurane via face mask to reach our set end-points. The patient’s temperament and behaviour may affect the outcome of both sedation and recovery. In several animal species, as well as in children, uncooperative and anxious patients are reported to experience sedation failure and emergence agitation [20,21,22]. In our case, SG4 exhibited signs of aggression and distress at presentation, which might be the reason for not achieving a surgical anaesthetic plane with our protocol. Dexmedetomidine competes with noradrenaline at the level of the alpha-2 adrenoreceptors; therefore, high circulating catecholamines would reduce dexmedetomidine’s clinical effect [23]. Furthermore, a high level of circulating catecholamines could have caused peripheral vasoconstriction, leading to a delay in its absorption after SC administration.

Despite failing to achieve a good quality of anaesthesia, this protocol provided a marked isoflurane-sparing effect. The isoflurane vaporizer was, in fact, set at 1.5% during induction and reduced to 1% during maintenance with a fixed fresh gas flow of 0.5 L/min. These values are considerably lower than the ones reported in the literature and used in clinical practice (5% isoflurane for induction and 2–3% isoflurane for maintenance with 1 L/min fresh gas flow) when a premedication with midazolam and buprenorphine is administered to perform orchiectomy [2].

Monitoring blood pressure in sugar gliders is not routinely performed due to their small size and the lack of validated methods and technology. Non-invasive blood pressure (NIBP) has not been validated in this species and the lack of cuffs of adequate size would cause the underestimation of systemic blood pressure leading to errors in interpretation and treatment [24]. The use of Doppler technology has been suggested in emergency settings to assess the blood pressure trend in critical patients [25]; however, no information is available as whether the reading obtained might reflect systolic or mean arterial pressure. Unfortunately, in our clinical setting, the Doppler monitor did not provide consistent blood pressure readings; therefore, the authors chose not to include this parameter in the case series.

Intubation is technically challenging in sugar gliders, thus end-tidal carbon dioxide and isoflurane concentrations were not available. As a result, the administration of high-dose halogenated agents on the cardiovascular system could not be fully evaluated, and titration of the inhalant anaesthetic was based solely on clinical assessment [2]. During elective surgical procedures, reducing or eliminating the use of inhalant anaesthetics may help prevent dose-dependent cardiovascular side effects—such as hypotension and decreased myocardial contractility—while also minimising personnel occupational exposure [26].

The dose of ketamine suggested in sugar gliders is between two and ten times higher than the one used in the present case series [2,27]. The onset and the duration of sedation when 10 to 50 mg/kg of ketamine is administered alone or in combination with other sedatives has not been previously reported. In the authors’ experience, recoveries are usually long, and reversal of the co-administered drugs is frequently required to accelerate recovery. This often results in emergence delirium and nausea. In the present case series, the use of a lower dose of ketamine could have contributed to the smooth and fast recovery observed in 3 out of 4 patients after atipamezole administration. The poor recovery exhibited by SG4 might be related to isoflurane administration, but we cannot exclude ketamine-related dysphoria. Ketamine is known to cause dose-dependent apneustic breathing [28]. In this case series, no apnoea or alteration of the respiratory pattern was observed, and hypoxaemia (SpO_2_ < 90%) [29] did not occur in any of the patients.

Castration surgery is considered to cause mild to moderate pain in animals [30], and it appears to be acute, inflammatory, visceral and somatic in nature.

In this case series, multimodal perioperative analgesia was provided administering both systemic and locoregional analgesics.

Dexmedetomidine and ketamine have analgesic properties, modulating visceral and somatic pain [12,31,32]. Ketamine, a N-methyl-D-aspartate (NMDA) antagonist, acts on several receptors and descending inhibitory pathways, leading to analgesia and prevention of wind up and pain facilitation associated with surgeries [33].

Dexmedetomidine inhibits nociceptive neurotransmission through the dorsal horn of the spinal cord, inhibits the release of norepinephrine at the level of the presynaptic membrane and promotes the release of acetylcholine from spinal interneurons, leading to pain regulation and analgesia [11].

Meloxicam, an NSAID that selectively inhibits cyclo-oxygenase 2, targets surgical inflammatory pain. In humans, preoperative administration is reported to cause pre-emptive analgesia and reduce postoperative pain compared to postoperative administration, reducing the need for opioid-based rescue analgesia [34]. In cats, the administration of meloxicam before ovariohysterectomy was associated with a lower pain score and better outcome compared with cats receiving preoperative buprenorphine [35]. Healthy koalas and other specialised foliage-eating marsupials showed high hepatic clearance of meloxicam compared to dogs and rats, which is likely dietary-related and might have an impact on the clinical doses administered in these species [36,37]. The pharmacokinetic profile of meloxicam in sugar gliders has not been studied yet but considering the role of inflammation in perioperative pain management, we decided to incorporate the use of meloxicam in our protocol as the NSAID routinely used in clinical practice at the current recommended dosage [2].

The intratesticular administration of lidocaine is a widespread locoregional technique in veterinary medicine described in several species [38,39,40], including sugar gliders [1,2]. Locoregional anaesthesia plays a fundamental role in pain management as it prevents the painful stimulus from being transmitted to the CNS [41]. The combination of systemic and locoregional analgesia provided in this case series appeared adequate to provide both antinociception during the procedure, as no increase in HR and/or RR was observed during the surgery, and postoperative pain relief. In all sugar gliders, food intake was resumed within an hour from the end of the procedure and no signs of discomfort (normal gait and posture, showing curiosity towards their surroundings and when offered food) were shown after one hour from the end of the surgery. Three out of four subjects allowed gentle manipulation and assessment of the surgical site without reaction. SG4 presented signs of dysphoria immediately after reversal and resumed his fractious demeanour afterwards.

Sugar gliders are a nocturnal prey species and tend not to display obvious antalgic behaviour when in pain [2]. Physiological variables (HR, RR, blood pressure) can be difficult to assess in these small-size patients when awake [2]. In addition, they appear to be aspecific indicators of pain as they can be influenced by stress and anxiety [2,42]. A dedicated pain scale has not yet been formulated in sugar gliders. Postoperative pain assessment was based on behaviours that we usually assess in our clinical practice to rule out signs of pain and discomfort in this species (stiff posture, avoidance behaviour, self-mutilation), together with more objective physiological data such as the return to normal bodily functions (eating, drinking, defecation and urination).

The poor recovery observed in SG4 suggests that the reversal of dexmedetomidine may need to be tailored to the quality of induction and the individual temperament of the animal. Reducing the dose of the antagonist could potentially result in a smoother and calmer recovery. On the other hand, the attempt to remove the stitches may indicate the presence of pain, as sugar gliders are known to self-mutilate when experiencing stress or discomfort [2]. However, this postoperative behaviour could also be triggered by the presence of sutures or skin glue [2]. We decided to allow full recovery before providing additional analgesia to rule out dysphoria and better assess the degree of discomfort or pain. One hour after the end of the surgery, the buster collar was removed and SG4 did not try to groom the surgical area and started to eat the food offered. To avoid possible side effects such as sedation, delay of food intake and nausea associated with opioid administration in pain-free animals, the authors decided to withhold buprenorphine administration.

## 5. Conclusions

Dexmedetomidine in combination with low-dose ketamine administered SC seemed to be a suitable protocol to perform orchiectomy in the sugar gliders of this case series, eliminating or reducing the need for isoflurane administration. Multimodal analgesia combining both systemic and locoregional techniques appeared to be sufficient to prevent nociception and postoperative pain. Temperament should be considered when choosing the anaesthetic protocol as it might affect the degree of sedation and quality of recovery.

## Figures and Tables

**Table 1:** Age, weight, mean physiological values and times (in minutes) recorded in 4 entire male sugar gliders (SG) undergoing castration.

	SG 1	SG 2	SG 3	SG 4
Age (months)	20	20	24	18
Weight (g)	73	70	80	56
Mean HR after induction (beats per minute)	210	200	180	220
Mean HR during surgery (beats per minute)	160	160	150	160
Mean RR during preparation (breaths per minute)	25	24	22	26
Mean RR during surgery (breaths per minute)	20	18	20	22
Surgery time (minutes)	6	7	5	7
Recovery time (minutes)	1	2	6	7
Overall time of the procedure (minutes)	11	12	11	19

## Data Availability

The original contributions presented in this study are included in the article. Further inquiries can be directed to the corresponding author.
